# Crown-Like Structures in Breast Adipose Tissue: Early Evidence and Current Issues in Breast Cancer

**DOI:** 10.3390/cancers13092222

**Published:** 2021-05-06

**Authors:** Maret L. Maliniak, Jasmine Miller-Kleinhenz, Deirdre P. Cronin-Fenton, Timothy L. Lash, Keerthi Gogineni, Emiel A. M. Janssen, Lauren E. McCullough

**Affiliations:** 1 Department of Epidemiology, Rollins School of Public Health, Emory University, Atlanta, GA 30322, USA; jmill37@emory.edu (J.M.-K.); timothy.lee.lash@emory.edu (T.L.L.); lauren.mccullough@emory.edu (L.E.M.); 2 Department of Clinical Epidemiology, Aarhus University Hospital, 8200 Aarhus, Denmark; dc@clin.au.dk; 3 Glenn Family Breast Center, Winship Cancer Institute of Emory University, Atlanta, GA 30322, USA; keerthi.gogineni@emory.edu; 4 Department of Hematology and Medical Oncology, Emory University School of Medicine, Atlanta, GA 30322, USA; 5 Department of Pathology, Stavanger University Hospital, 4011 Stavanger, Norway; emilius.adrianus.maria.janssen@sus.no

**Keywords:** crown-like structures, obesity, inflammation, breast cancer

## Abstract

**Simple Summary:**

Obesity increases the risk of postmenopausal, hormone receptor-positive breast cancer and has been linked to a higher risk of recurrence and mortality. During obesity, adipose tissue can become dysfunctional, resulting in chronic low-grade inflammation. Crown-like structures in breast adipose tissue (CLS-B), composed of macrophages surrounding dead or dying adipocytes in a crown-like pattern, are a new histologic marker of local inflammation. In this review, we aim to evaluate the early evidence of CLS-B in breast cancer. There is consistent evidence that CLS-B are more frequently detected among obese compared to non-obese breast cancer patients. Additionally, several studies have found that CLS-B presence is associated with metabolic and inflammatory factors that contribute to breast cancer development and progression. However, more studies are needed to understand the potential clinical utility of CLS-B as a marker of breast cancer risk or prognosis.

**Abstract:**

Obesity is an established risk factor for postmenopausal breast cancer and has been linked to worse breast cancer prognosis, most clearly for hormone receptor-positive breast cancers. The underlying mechanisms of the obesity–breast cancer association are not fully understood, but growing evidence points to the breast adipose tissue microenvironment playing an important role. Obesity-induced adipose tissue dysfunction can result in a chronic state of low-grade inflammation. Crown-like structures of the breast (CLS-B) were recently identified as a histologic marker of local inflammation. In this review, we evaluate the early evidence of CLS-B in breast cancer. Data from preclinical and clinical studies show that these inflammatory lesions within the breast are associated with local NF-κB activation, increased aromatase activity, and elevation of pro-inflammatory mediators (TNFα, IL-1β, IL-6, and COX-2-derived PGE_2_)—factors involved in multiple pathways of breast cancer development and progression. There is also substantial evidence from epidemiologic studies that CLS-B are associated with greater adiposity among breast cancer patients. However, there is insufficient evidence that CLS-B impact breast cancer risk or prognosis. Comparisons across studies of prognosis were complicated by differences in CLS-B evaluation and deficiencies in study design, which future studies should take into consideration. Breast adipose tissue inflammation provides a plausible explanation for the obesity–breast cancer association, but further study is needed to establish its role and whether markers such as CLS-B are clinically useful.

## 1. Introduction

Obesity is an established risk factor for postmenopausal breast cancer [1,2] and has been linked to a higher risk of recurrence and mortality [1,3,4]. Various mechanisms have been proposed by which obesity affects breast cancer. Broadly, these include hormonal effects and chronic inflammation occurring at both the systemic and local levels [5]. Most epidemiologic studies have examined these mechanisms by measuring systemic levels of sex hormones and inflammatory markers. Less is known about the local, tissue-level effects, which are thought to be distinct and better related to the carcinogenic process [6]. Crown-like structures in the breast adipose tissue (CLS-B) are a histologic marker of local inflammation that may provide biologic insight into the obesity–breast cancer association [7,8]. Below, we briefly review the relation between obesity and breast cancer risk and prognosis; summarize the current epidemiological findings for the role of CLS-B in breast cancer; and conclude with possible future directions for research in this topic.

## 2. The Complex Relationship between Obesity and Breast Cancer

The association between obesity, most commonly measured using body mass index (BMI), and breast cancer risk is complex. It depends on the age that obesity is assessed, menopausal status at diagnosis, and hormone receptor (HR) status—as discussed in several reviews [9,10,11]. According to the most recent meta-analysis by the World Cancer Research Fund International Continuous Update Project (WCRF CUP), elevated BMI in early adulthood (~18–30 years) is associated with a lower risk of both premenopausal (per 5 kg/m^2^; summary RR: 0.86, 95%CI: 0.78 to 0.96) and postmenopausal breast cancer (per 5 kg/m^2^; summary RR: 0.81, 95%CI: 0.75 to 0.87) [11]. Being overweight or obese as an adult before menopause is also inversely associated with premenopausal breast cancer risk (per 5 kg/m^2^ increase in BMI; summary RR: 0.94, 95%CI: 0.91 to 0.98) [11] although some studies suggest that this inverse association is limited to HR-positive tumors [12,13,14]. There is emerging evidence of null or positive associations among premenopausal women with HR-negative tumors, particularly triple-negative breast cancers (TNBC) [14,15,16,17]. In contrast, elevated BMI in adulthood is consistently related to a higher risk of postmenopausal breast cancer—with the association limited to HR-positive tumors (per 5 kg/m^2^; summary RR: 1.18, 95%CI: 1.10 to 1.27) [11].

When other adiposity measures are considered, particularly indicators of central adiposity, positive associations are observed for postmenopausal breast cancers that are generally limited to HR-positive tumors and attenuate after adjusting for BMI [18,19,20,21]. Associations differ for premenopausal breast cancers. For example, waist circumference is positively associated with premenopausal breast cancer risk when adjusting for BMI (per 10 cm; summary RR: 1.15, 95%CI: 1.05 to 1.26) but not without BMI adjustment (per 10 cm; summary RR: 0.99, 95%CI: 0.94 to 1.03) [11]. Associations for waist-to-hip ratio (WHR) similarly become positive when adjusting for BMI [11], providing compelling evidence that the localization of fat depots is important in understanding premenopausal breast cancer risk. Emerging evidence has also shown that central adiposity is more strongly associated with estrogen receptor (ER)-negative/progesterone receptor (PR)-negative tumors than ER-positive/PR-positive tumors for premenopausal breast cancers [18,19,20]. These results suggest that estrogen-independent mechanisms such as inflammation [22,23] and insulin signaling pathways [24,25] may play a larger role for premenopausal breast cancers. Increased central adiposity is characterized by increased visceral adipose tissue, which is more metabolically active, insulin resistant, and contains more inflammatory and immune cells than subcutaneous adipose tissue [26,27].

Jiralerspong and Goodwin reviewed the role of obesity on breast cancer prognosis [1]. The authors reported that obesity is associated with a 35% to 40% increased risk of breast cancer recurrence and death regardless of menopausal or HR status [1]. This conclusion was based on three large meta-analyses of breast cancer mortality [3,4,28] and five epidemiologic studies of recurrence [29,30,31,32,33], with positive associations observed for distant recurrence. The WCRF CUP meta-analysis estimate for breast cancer mortality, comparing the highest versus lowest BMI categories, is consistent with their conclusions (summary RR: 1.35, 95%CI: 1.23 to 1.48) [34]. Although there is limited evidence of heterogeneity by HR status, meta-analyses and epidemiologic investigations show stronger and more consistently positive associations for ER-positive than ER-negative breast cancers. A recent meta-analysis of nine studies found no association between obesity and disease-free survival among triple-negative breast cancer (TNBC) patients (≥30 versus <30 kg/m^2^: summary RR = 0.98, 95%CI: 0.77 to 1.24) [35]. Various reasons could explain the mixed results for ER-negative cancers such as TNBC, including heterogeneity within the ER-negative and TNBC molecular subtypes or heterogeneity in study populations and methods [1]. Further research is needed to clarify associations among ER-negative patients.

The complex relationship between obesity and breast cancer points to multiple pathways by which obesity is linked to breast cancer—most thought to be risk promoting, but some of which might be risk reducing either directly or indirectly. Various mechanisms have been proposed as described in previous reviews [36,37,38,39]. To date, much of the epidemiologic evidence is limited to studies examining the systemic effects of adiposity. However, recent research is shifting towards exploring the breast adipose tissue microenvironment as a driver of carcinogenesis, which may be unique from other adipose depots [40].

### 2.1. Obesity-Induced Changes in Adipose Tissue 

Obesity is characterized by adipose tissue expansion [22]. Adipose tissue was previously thought to be inert and primarily for energy storage, but it is now recognized as one of the body’s largest endocrine organs, capable of secreting >50 adipokines, cytokines, and chemokines [22]. Adipose tissue is comprised of adipocytes but also includes preadipocytes, fibroblasts, macrophages, lymphocytes, pericytes, extracellular matrix, and endothelial cells [41]. Expansion of adipose tissue, specifically white adipose tissue (WAT), during periods of weight gain results in adipose tissue dysfunction and inflammation. The consequences of obesity-induced adipose tissue dysfunction have been summarized in a previous review by Picon-Ruiz et al. (see Figure 4 of the review by Picon-Ruiz et al.) [37]. They include increased secretion of pro-inflammatory adipokines and cytokines such as leptin, interleukin (IL)-1β, IL-6, and tumor necrosis factor (TNF)-α, as well as a decrease in adiponectin, an anti-inflammatory adipokine [22]. This pro-inflammatory state stimulates lipolysis and secretion of free fatty acids that can subsequently induce insulin resistance, leading to higher circulating levels of insulin and insulin-like growth factors (IGFs) [22].

Importantly for breast cancer, aromatase—the rate-limiting enzyme for estrogen biosynthesis—is stimulated in the adipose tissue by adipose-derived factors (IL-1β, IL-6, TNF-α, and prostaglandin (PG) E_2_) as well as liver-derived IGF-1 [22]. Moreover, increased insulin associated with overnutrition reduces hepatic production of sex hormone-binding globulin (SHBG), which binds to estradiol. This combination of increased aromatization in adipose tissue and lower SHBG to bind to estradiol results in increased estrogen production and bioavailability. After menopause, adipose tissue becomes the main source of local and circulating estrogens, the primary driver of ER-positive cancers among postmenopausal women. There is substantial epidemiologic evidence demonstrating that circulating estrogens, namely free estradiol, mediate the association between BMI and postmenopausal ER-positive breast cancer [42,43,44]. Furthermore, the increased risk of postmenopausal ER-positive breast cancer associated with higher BMI is not observed among women taking exogenous sex hormones who have elevated levels of circulating estrogens regardless of BMI [45].

There is also a shift in the adipose tissue immune cell landscape with obesity, leading to an increase in macrophages, particularly of the pro-inflammatory phenotype. WAT in lean individuals contains 10–15% macrophages that tend to be polarized toward the anti-inflammatory M2 phenotype, which is associated with adipose tissue metabolic homeostasis and insulin sensitivity [46]. In contrast, WAT in obese individuals contains 50–60% macrophages that tend to be polarized toward the pro-inflammatory M1 phenotype. M1 macrophages produce pro-inflammatory cytokines that contribute to sustained adipose inflammation [46]. Additionally, as adipocytes undergo hypertrophy, some outgrow their blood supply, leading to hypoxia and activating hypoxia-inducible factor-1 (HIF-1). HIF-1 upregulates leptin and vascular endothelial growth factor (VEGF) and inhibits adiponectin—factors that can promote the invasion and metastasis of cancer [37]. Hypoxia can also lead to adipocyte stress and death. Macrophages surround dead or dying adipocytes in a crown-like pattern, thus presenting pathologically as crown-like structures (CLS). CLS-associated macrophages intensely produce pro-inflammatory mediators and are beginning to be recognized as a hallmark of WAT inflammation [22]. In addition to cytokine overproduction, CLS in gluteal and abdominal fat have been associated with altered adipose tissue gene expression, systemic insulin resistance, and vascular endothelial dysfunction [47,48].

Adipocytes and macrophages, such as those that form CLS, produce numerous pro-inflammatory factors. These factors promote metabolic dysregulation and chronic low-grade inflammation within the adipose tissue microenvironment, thereby contributing to the development of chronic hyperinsulinemia, insulin resistance, dyslipidemia, and oxidative stress [22]. These conditions may be associated with cancer progression via stimulating cell proliferation, loss of epithelial integrity, angiogenesis, cell migration, and metastasis [22].

### 2.2. Breast Adipose Tissue Microenvironment

The role of the breast adipose tissue microenvironment in breast cancer development and progression is a relatively new area of research. As adipose tissue is a major component of the breast, breast cancers originate within an adipose-rich microenvironment. However, the biology of the breast adipose depot and how it changes during obesity are incompletely understood. There is known diversity in adipose depots [40], most notably between subcutaneous WAT and visceral WAT—the latter considered more metabolically active and strongly related to obesity-related comorbidities such as type 2 diabetes and cardiovascular disease [27,49]. Growing scientific evidence shows obesity-related conditions such as insulin resistance, type 2 diabetes [50,51], and the metabolic syndrome [52] as well as obesity-related systemic markers (e.g., leptin [53], adiponectin [54], SHBG [55], insulin and the IGF axis [56,57,58,59,60] and C-reactive protein [61,62]) are associated with breast cancer. How breast adipose tissue differs from other adipose depots is not fully elucidated, but it does have unique tissue-specific functions [40]. Adipose cells in the breast continually interact with breast epithelial cells by providing structural support and regulating signals [40]. In vitro and in vivo studies show that breast adipocytes are required for initial growth, branching, and maintenance of the epithelial ducts as well as functional differentiation before pregnancy [40]. Additionally, the mammary gland epithelium and adipose tissue both undergo cyclic remodeling during pregnancy, lactation, and involution [40]. The role of adipose tissue in normal mammary gland development has been proposed as a possible explanation of the inverse association observed between obesity and premenopausal breast cancer [63]. Other explanations include disruptions to the menstrual cycle [64] and lower mammographic density [65,66] in obese premenopausal women.

Interactions between adipose tissue and breast epithelium may also play a role in pathological conditions of the breast such as breast cancer. For instance, emerging evidence suggests breast adipose tissue shares similar pro-inflammatory mechanisms as other adipose tissue depots including obesity-induced macrophage infiltration [22]. The pioneering work of Dannenberg and colleagues demonstrated that macrophage infiltration, as evidenced by the presence of CLS, occurs in the adipose tissue of the mouse mammary gland and in the breast of humans [67]. These crown-like structures of the breast (CLS-B) have garnered considerable interest as a histologic marker of local WAT inflammation. Given the distinct features and functions of breast adipose tissue and the close proximity to where breast cancers originate, these localized inflammatory lesions may provide unique insight into the underlying mechanisms of the obesity–breast cancer association. The rest of this review will focus on CLS-B and summarize the current epidemiologic evidence regarding their role in breast cancer development and prognosis.

## 3. Crown-Like Structures of the Breast (CLS-B): Histologic Marker of Local Inflammation 

**Figure 1:**
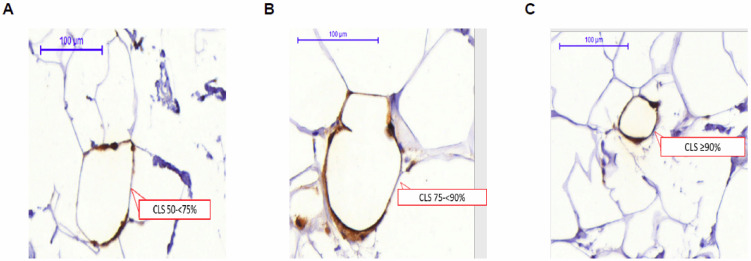
Whole slide digital images of anti—CD68-immunostained breast tissue captured with 3DHISTECH Panoramic Scanner 150 and analyzed using Panoramic Viewer 1.15.4 (3DHISTECH Ltd., Budapest, Hungary) from a case with CLS-B (**A**) CD68-immunostained tissue showing 50–< 75% adipocyte encirclement; (**B**) CD68-immunostained tissue showing 75–< 90% adipocyte encirclement; (**C**) CD68-immunostained tissue showing ≥90% adipocyte encirclement. Adapted from Maliniak et al. 2020 [69].

Similar to CLS formation in other adipose depots, CLS-B are thought to form during weight gain when adipocyte hypertrophy occurs in the breast, leading to adipocyte stress and death, macrophage recruitment, and encirclement of necrotic adipocytes by macrophages in a crown-like pattern [68]. There is currently no standardized methodology for assessing CLS-B in epidemiologic studies (as discussed later in this review). Generally, CLS-B are identified by staining breast adipose tissue for a macrophage marker (e.g., CD68) using immunohistochemistry. Adipocyte encirclement by macrophages may vary from partial to complete encirclement (Figure 1). No minimum amount of encirclement has been proposed for defining CLS-B. One study used ≥50% encirclement for primary analyses and made comparisons using higher cut points (≥75% and ≥90%) with few differences in conclusions by cut point used [69]. Based on the study’s definition of CLS-B, a breast pathologist then enumerates CLS-B typically by visual assessment. Patient breast adipose tissue samples are often classified according to the presence or absence of CLS-B and can be further quantified using the density of CLS-B per total area of breast WAT (e.g., CLS-B/cm^2^ of WAT). Previous studies have used CLS-B density for assessing the severity of WAT inflammation in the breast [69,70,71,72,73]. These studies used the median density value among those with CLS-B to categorize the severity of WAT inflammation with those below the median considered as having mild WAT inflammation and those above the median as having severe WAT inflammation [69,70,71,72,73]. Thus, cut points differ by study and no empirically-based threshold has been determined for defining clinically relevant severe WAT inflammation.

**Figure 2:**
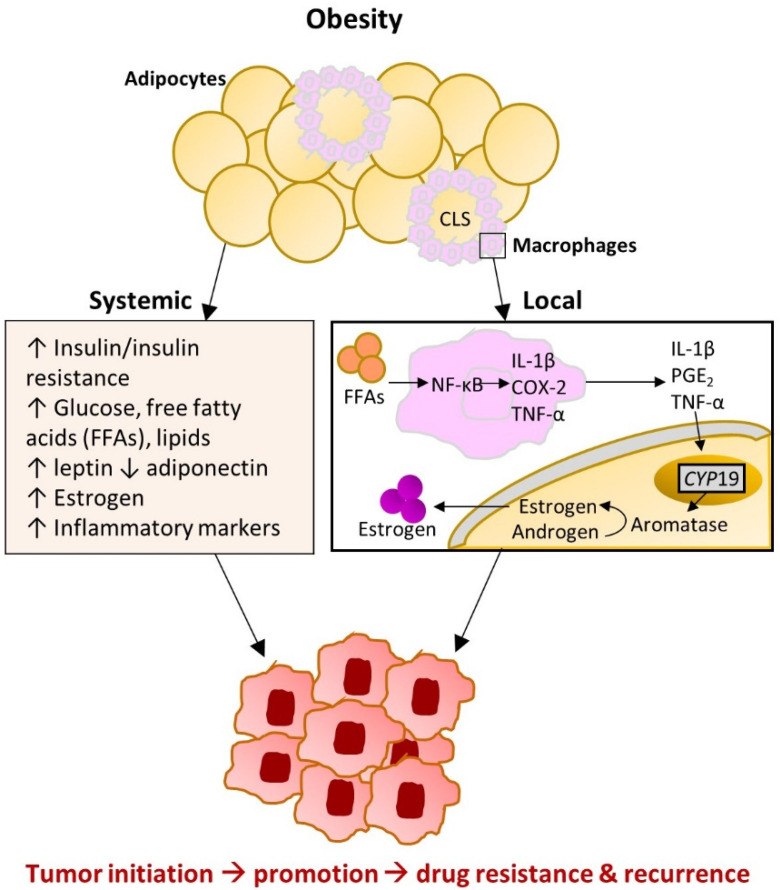
Hormonal and inflammatory effects of obesity-induced adipose dysfunction occurring at both the systemic and local levels. Abbreviations: CLS: crown-like structure; COX-2: cyclooxygenase-2; FFAs: free fatty acids; IL: interleukin; NF-κB: nuclear factor kappa B; PGE2: prostaglandin E2; TNF-α: tumor necrosis factor alpha. Adapted with permission of Annual Reviews, Inc., from Iyengar NM, Hudis CA, Dannenberg AJ. Obesity and cancer: local and systemic mechanisms. Annu Rev Med. 2015; 66: 297–309; permission conveyed through Copyright Clearance Center, Inc. [74].

Initial studies of CLS-B suggest that they create a microenvironment in the breast that is rich in pro-inflammatory cytokines and increased aromatization (Figure 2) [67,74]. This occurs through macrophage exposure to saturated fatty acids from lipolysis, which can activate Toll-like receptor 4 (TLR4) at the macrophage cell surface, leading to activation of the transcription factor nuclear factor kappa B (NF-κB) [75]. A small pilot study (n = 30) found that CLS-B presence was associated with elevated NF-κB binding activity in the breast tissue of women who received mastectomy to prevent or treat breast cancer [67]. NF-κB regulates the expression of more than 500 genes involved in inflammation, cellular transformation, survival, proliferation, angiogenesis, metastasis, and chemo- and radiotherapy resistance [76]. Some of these include TNFα, IL-1β, IL-6, and COX-2-derived PGE_2_, which are related to increased aromatase activity in adipose tissue as well as other protumorigenic mechanisms [67,73,77,78,79]. The same pilot study and two other studies found that CLS-B were positively associated with aromatase activity in breast tissue [67,71,72]. One of these studies was limited to ideal-weight women (BMI < 25 kg/m^2^), suggesting that local inflammation and increased aromatization (as indicated by CLS-B presence) occurs even in the absence of obesity. Another study conducted among 83 postmenopausal breast cancer patients found that CLS-B were associated with increased ratios of estrogens to androgens in breast fat and serum, indicative of increased aromatization with local and systemic effects [80].

Furthermore, CLS-B have been associated with an unfavorable metabolic and inflammatory profile. Four studies among mostly breast cancer patients demonstrated associations between CLS-B and circulating metabolic and inflammatory markers [71,81,82,83]. In an analysis of 100 women who underwent mastectomy and donated fasting blood specimens at the time of surgery, CLS-B presence was associated with elevated levels of glucose, insulin, leptin, triglycerides, high-sensitivity C-reactive protein (CRP), and IL-6; CLS-B presence was associated with lower levels of HDL cholesterol and adiponectin [82]. Similar findings were observed in the three other studies [71,81,83]. None of these studies adjusted for BMI in their analyses; however, one study was limited to ideal-weight women, which provides some evidence that the observed associations were not entirely driven by elevated BMI among women with CLS-B [71].

Further description of the molecular pathways between CLS-B and sustained local WAT inflammation can be found in several reviews [7,23,36,75,78].

## 4. Potential Etiologic Drivers of CLS-B

Epidemiologic evidence examining the role of CLS-B in breast cancer is sparse. Table 1 provides a summary of previous epidemiologic studies (*n* = 14). The majority of these were cross-sectional analyses aimed at identifying factors related to CLS-B, as reviewed below.

### 4.1. Obesity and CLS-B

Cross-sectional studies have consistently demonstrated a strong, positive association between adiposity, typically measured using BMI, and CLS-B presence and density in numerous independent populations of predominantly breast cancer patients (Table 2) [73,80,81,83,87]. For example, one study including 237 patients who underwent mastectomy for breast cancer prevention or treatment, detected CLS-B in 90% of obese patients (BMI ≥ 30 kg/m^2^), 53% of overweight patients (BMI 25–< 30 kg/m^2^), and 34% of ideal-weight patients (BMI < 25 kg/m^2^) [73]. The relatively high proportion of ideal-weight women with CLS-B suggests that even among lean women, adipocyte hypertrophy may be relevant for breast carcinogenesis [71].

Few studies have examined the association between CLS-B and other measures of adiposity. A Norwegian study including 107 patients with early-stage breast cancer examined associations between CLS-B and three adiposity measures (BMI, WHR, and percent truncal fat) [83]. It found that women with a BMI ≥ 30 kg/m^2^ were almost seven times as likely to have CLS-B present as women with a BMI < 25 kg/m^2^, although the association was imprecise as demonstrated by the wide 95% confidence interval (OR: 6.9; 95%CI: 1.35 to 35.0) [83]. Strong associations were also found for WHR (>0.85 vs. ≤0.85; OR: 3.26, 95%CI: 1.35 to 7.85) and percent truncal fat (per standard deviation; OR: 3.58, 95%CI: 2.00 to 6.44). These associations were generally consistent across menopausal status except for percent truncal fat, which showed a stronger association among premenopausal women [83]. Another study with body composition measurements among Taiwanese breast cancer patients found that women with CLS-B had a higher body fat percentage (33% vs. 27.5%; *p* < 0.01), greater visceral (2.7 kg vs. 1.5 kg; *p* < 0.01), and subcutaneous adipose tissue (20.1 kg vs. 14 kg; *p* < 0.01) compared with patients without CLS-B [81].

At the tissue level, several studies have demonstrated that CLS-B are positively correlated with breast adipocyte diameter, as would be expected given their association with obesity [67,69,70,71,73,81,83,84]. Even among ideal-weight women, a positive, linear association was found between CLS-B density and adipocyte diameter (ρ = 0.36; *p* < 0.01) [71].

There is little evidence relating obesity with CLS-B in study populations of women without breast cancer due to the difficulty in obtaining breast adipose tissue for such analyses. In one case–control study including patients with benign breast disease (BBD) that was nested within the Mayo BBD cohort and also included a group of women that donated normal breast tissue to the Komen Normal Tissue Bank (KTB), CLS-B were detected in 7% of women with BMI < 25 kg/m^2^, 13% with BMI 25–29, and 29% with BMI ≥ 30 (*p* = 0.0005) [85]. This association was primarily driven by the women with BBD—as only 3% of the KTB donors were found to have CLS-B present in their tissue samples [85]. In another case–control study including African American women from the Detroit BBD Cohort and African American KTB donors, the authors reported no association between BMI and CLS-B [84].

### 4.2. Other Factors and CLS-B

Few clinical and lifestyle factors beyond BMI have been identified as associated with CLS-B (Table 3). In several investigations, older age and postmenopausal status have been linked to higher prevalence of CLS-B detection [69,70,73,80,86]. This relation could be due to increased breast fat percentage [88] and/or alterations to adipose tissue [89] that occur with age and menopausal transition. One study compared 102 premenopausal and 59 postmenopausal women who underwent mastectomy for breast cancer treatment or prevention and found that postmenopausal women were more likely to have a higher BMI, larger breast adipocytes, severe breast WAT inflammation as measured by the number of CLS-B/cm^2^, and greater local aromatase activity than premenopausal women [72].

Race/ethnicity has also been explored as a possible driver of CLS-B due to the known differences in the prevalence of obesity, obesity-related comorbidities, and visceral fat accumulation by race/ethnicity [90]. Evidence of greater CLS-B density among Black breast cancer patients was observed in two studies [69,87]; however, the association attenuated after BMI adjustment in one investigation [69]. The other study did not obtain patient-level BMI data. Other research shows no evidence of differences in CLS-B detection by country of origin among Hispanic/Latina patients [70], or when comparing Taiwanese to US Caucasian patients [81]. Notably, the positive associations between BMI and CLS-B were consistent across these various racial/ethnic groups [69,81].

Associations with other patient factors (e.g., smoking status, reproductive factors, family history of breast cancer) and tumor characteristics are largely inconclusive due to the scarcity of evidence for these other factors.

## 5. CLS-B and Incident Breast Cancer

There have been two studies examining the association between CLS-B and breast cancer incidence (Table 4), both case–control studies nested within BBD cohorts [84,85]. The first, conducted in the Mayo BBD cohort—a large cohort of women with biopsy-proven BBD—included 86 cases (BBD patients with breast cancer) and 86 age-matched controls (BBD patients without breast cancer) [85]. CLS-B were assessed in the index BBD biopsy (before breast cancer diagnosis for cases) and found to be more frequent among cases (24%) than controls (19%). The association was most robust when examining >5 CLS-B/sample (adjusted OR: 6.8, 95%CI: 1.4 to 32.4) [85]. The second investigation included 55 cases and 47 controls nested within the Detroit BBD cohort [84]. All women were African American. This study similarly found more frequent CLS-B among cases (67%) compared with controls (40%), and a strong association for ≥5 CLS-B/sample (adjusted OR: 4.99, 95%CI: 1.32 to 18.9) [84]. There are several limitations to these studies as both had small sample sizes, resulting in a large amount of uncertainty in the effect estimates. In both studies, models adjusted for a small number of potential confounders (e.g., adipose area, histologic impression, and BMI in one study [85]). Finally, since these studies were both conducted among women with BBD, it is unknown whether the results can be generalized to women without BBD.

## 6. CLS-B as a Potential Driver of Prognosis

Four studies included follow-up data to examine the impact of CLS-B detection at diagnosis on clinical outcomes (Table 4) [69,82,86,87]. Results have been inconsistent and conclusions limited due to lack of power/precision, varying study methods, and differences in study populations. Iyengar et al. conducted the first study examining CLS-B and prognosis in a study population of patients that all developed distant recurrence. CD68 (a pan-macrophage marker) was used to identify CLS-B in non-tumor-containing breast adipose tissue. In this cohort, CLS-B presence was associated with shortened average time to distant recurrence (adjusted HR: 1.83, 95%CI: 1.07 to 3.13) [82]. However, because this was a case-only analysis in which selection was based on having developed the outcome of interest, interpretability and generalizability of results are limited [91]. The second study by Koru-Sengul et al. assessed CLS-B using three macrophage markers for detection in the fat portions of tumor-containing tissue (CD206 = M2 marker, CD40 = M1 marker, and CD163 = pan-macrophage marker) [87]. They observed positive associations with worse overall survival for density of CLS detected by CD40 (adjusted HR: 9.14, 90%CI: 1.00 to 83.60) and CD163 (adjusted HR: 2.14, 95%CI: 0.46 to 9.96) but not CD206 (adjusted HR: 0.65, 90%CI: 0.03 to 12.58) although all associations were very imprecise [87]. Associations for progression-free survival were similarly imprecise but positive regardless of macrophage marker (e.g., for CD40: adjusted HR: 4.21, 90%CI: 0.49 to 34.92) [87]. A study by Cha et al. used CD68 and CD163 (described as an M2 macrophage marker in this study) to detect CLS-B in three groups of patients with breast adipose tissue (Group 1 from reduction mammoplasty; Group 2 from non-neoplastic tissue from resected breast cancer specimens; Group 3 from breast cancer tissue specimens) [86]. However, this study had few recurrences (*n* = 18) and deaths (*n* = 11) among breast cancer patients and did not report effect estimates for associations between CLS-B and clinical outcomes [86]. In the largest study to date, we recently investigated this topic in a diverse cohort of Black and White breast cancer patients unrestricted to outcome and used CD68 to identify CLS-B in non-tumor-containing breast adipose tissue [69]. We found no evidence of an association between CLS-B with overall (adjusted HR: 1.02, 95%CI: 0.55 to 1.87) or progression-free survival (adjusted HR: 0.99, 95%CI: 0.59 to 1.67). Despite this being the most methodologically robust study to date, estimates remained imprecise and may have been biased toward the null due to misclassification of CLS-B; only one tissue specimen per patient was used for assessing CLS-B as compared to five tissue specimens per patient in the study by Iyengar et al. [69,82]. Given the limitations and scarcity of evidence, it is still unclear whether CLS-B could serve as a prognostic marker in breast cancer.

## 7. Future Directions

The role of CLS-B in breast cancer is not established. We have identified three broad areas for future work in this area: (1) establishing standards for CLS-B assessment; (2) assessing the role of CLS-B in breast cancer incidence, and (3) assessing the role of CLS-B on therapeutic effectiveness and breast cancer prognosis.

### 7.1. Methodology in CLS-B Assessment

Standards for CLS-B assessment based on empirical evidence are needed for a broader assessment in epidemiologic studies and to facilitate comparisons across studies. Different methods have been used across epidemiologic studies to date (Table 5). Questions need to be answered regarding the number of tissue specimens required to adequately assess CLS-B; the most clinically relevant macrophage marker(s) for identifying CLS-B; and whether CLS-B can be appropriately evaluated in tissue samples beyond those obtained from mastectomy.

The number of adipose tissue samples used for CLS-B evaluation influences how many participants are classified as having CLS-B present (assuming the number of specimens examined positively correlates with the total area of adipocytes examined). Using only one tissue specimen per participant results in a lower proportion with CLS-B detected, as demonstrated in the studies by Maliniak et al. [69] and Mullooly et al. [80] where 30% and 36% of breast cancer patients were reported with CLS-B present. These proportions are lower than all of the studies that used 4–5 specimens per case, even the one study of ideal-weight women, where 39% of patients were classified with CLS-B [71]. It is likely that some subjects who truly have CLS-B are misclassified as not having CLS-B when using only one tissue specimen. In a small study of 30 patients, 14 (47%) had CLS-B present on ≥1 of 5 tissue slides examined per patient [67]. Of these 14 patients, seven had CLS-B present on a single slide, six had CLS-B present on 2–4 slides, and only one patient had CLS-B present on all slides [67]. This small study provides evidence that CLS-B is heterogeneous across breast adipose tissue samples; sensitivity might be low for detecting CLS-B by only using one sample. However, the cost, availability of tissue, and pathologist time spent assessing CLS-B on five specimens per participant might be prohibitive—especially for large-scale epidemiologic studies, which are needed to adequately assess the prognostic value of CLS-B. High-throughput assessment of CLS-B using novel imaging technologies [92] would decrease pathologist time but have not been used for detection of CLS-B. To balance cost and resources with potential misclassification, additional data evaluating the number of tissue specimens required for adequate CLS-B assessment are needed.

The antibody used for detecting CLS-B has varied across studies, with some using multiple markers. Although most previous investigations used the CD68 pan-macrophage marker, two studies employing macrophage markers specific to M1 and M2 phenotypes found differential associations with patient and tumor characteristics [86] as well as prognosis [87]. It is unknown whether differences detected by macrophage markers are biologically or clinically meaningful as they could have been due to chance in these relatively small studies. However, given the biologic differences between pro-inflammatory M1 and anti-inflammatory M2 macrophages, they could affect tumor biology differently [7,93]. CLS are thought to primarily consist of pro-inflammatory M1 macrophages since they are associated with a pro-inflammatory microenvironment [94]; however, both M1 and M2 macrophages increase in the adipose tissue microenvironment with obesity and their role in the tumor microenvironment differs. Anti-inflammatory M2 macrophages are considered protumorigenic as they express immunosuppressive molecules such as IL-10, programmed death-ligand 1 (PD-L1), and transforming growth factor (TGF)-β—favoring tumor growth. In contrast, M1 macrophages are considered antitumorigenic as they express pro-inflammatory, microbicidal cytokines [93,94]. It has been proposed that the ratio of M1/M2 macrophages may be a more biologically relevant indicator for cancer prognosis [93]. It is unclear how this emerging evidence of the diversity of the immune cell landscape might impact studies of CLS-B.

Finally, most studies among breast cancer patients have examined CLS-B in adipose tissue remote from the tumor—although a few have examined them more proximal to the tumor [83,86,87]. Tumor tissue is more widely available, especially for early-stage breast cancer patients who may opt for breast-conserving surgery, so evaluation of CLS-B in the surrounding adipose tissue would allow for assessment in a wider population. However, differences in CLS-B by proximity to the tumor are unclear. One study by Cha et al. found that CLS-B were present in 13–18% of tumor-containing tissue, but no CLS-B were detected in non-tumor-containing tissue [86]. Although this contrasts with several studies that utilized non-tumor-containing tissue from mastectomy and found up to 57% of patients with CLS-B [69,71,73,80,81,82], it does support the possibility that CLS-B are more frequent in close proximity to the tumor. No study has directly compared CLS-B associations with patient characteristics or prognosis by proximity to the tumor within the same study population. However, a study by Vaysse et al. examined CLS-B in adipose tissue surrounding the tumor primarily from lumpectomy specimens and found a strong association between obesity and CLS-B that was similar to associations found in studies where CLS-B were evaluated in non-tumor-containing tissue [83]. Nevertheless, studies should carefully consider assessment methods and their implications. CLS-B remote from the tumor may be more representative of inflammation that existed prior to breast malignancy while CLS-B in close proximity to the tumor could be a direct result of the tumor or diagnostic work-up (i.e., biopsy).

### 7.2. The Role of CLS-B in the Incidence of Breast Cancer

Assessing the role of CLS-B in breast cancer incidence is difficult, given that breast adipose tissue is not easily obtained or routinely collected. Obtaining tissue from normal breast tissue banks, reduction mammoplasty, or BBD cohorts provide opportunities to explore this association. Yet, substantial challenges remain. For instance, some studies suggest that the prevalence of CLS-B may be low among women without BBD or breast cancer (e.g., 3% and 18% in two samples of KTB normal breast tissue donors [84,85] and 2% in a sample of women who received reduction mammoplasty [86]). Thus, large study samples would be needed to adequately power these analyses. The greater detection of CLS-B among African American KTB donors found in one study (18% with CLS-B present) suggests that studies may be more feasible in this group [84]. Thus, far, no study has examined whether CLS-B are associated with the risk of certain molecular subtypes of breast cancer. ER-negative breast cancers, including TNBC, are more frequent in African American women and are thought to be related to activation of NF-κB and inflammatory pathways [76,95]. The potentially higher frequency of CLS-B among African American women, who also tend to have a higher BMI than their European ancestry counterparts, could indicate a biologic explanation for the observed association between obesity and TNBC [17]. This hypothesis has not been tested.

The current studies of CLS-B and breast cancer risk were conducted within BBD cohorts, which is an opportune study group given that CLS-B is more frequent among BBD patients (compared to women without BBD) and breast adipose tissue from biopsy is more easily accessible. Although results from these studies may not be generalizable to women without BBD, they could provide some biologic insight—especially if future studies explore differences by menopausal status and molecular subtype. There may also be important public health and clinical implications for this select group. It has been suggested that CLS-B could be used to identify BBD patients exhibiting metabolic obesity who might benefit from behavioral changes or more frequent breast cancer screening [84]. Prevention measures based on CLS-B status would not be practical outside of this group unless a less invasive biomarker associated with CLS-B is identified.

### 7.3. The Role of CLS-B in Predicting Therapeutic Effectiveness and Breast Cancer Prognosis

Since breast tissue is often obtained from breast cancer patients receiving surgery as part of their treatment, investigating the association between CLS-B and prognosis is more feasible and likely to have greater public health implications than uncovering any etiologic association. Based on current evidence, the role of CLS-B in breast cancer prognosis is unclear. Well-designed and well-powered studies are needed to determine the predictive and prognostic value of CLS-B.

Future studies should examine heterogeneity in associations by host and tumor characteristics as well as in treatments received. Given the proposed mechanisms of CLS-B and clinical evidence that obesity affects the pharmacokinetics and effectiveness of some chemo- and hormonal therapies [96,97,98,99,100,101,102,103], these lesions may affect certain subgroups of breast cancer patients more than others [1,104]. Adipocyte–tumor crosstalk is an active area of preclinical research, with some studies in breast and other cancers showing that adipocytes can promote chemoresistance through various mechanisms [105,106]. Moreover, macrophages may further protect tumor cells from the cytotoxic effects of chemotherapy—as suggested in several in vitro studies [7,107,108]. Thus, adipocytes and macrophages, such as those in CLS-B, might work together to reduce efficacy of cancer therapies. Future preclinical and clinical studies should further explore these potential interactions.

Given that CLS-B are associated with increased aromatase expression and activity, as supported by several animal and human studies [67,71,73,79,80], they may play a role in the efficacy of breast cancer therapies that target aromatase. While the evidence is mixed, some studies have shown aromatase inhibitors (AIs) but not tamoxifen (which targets the estrogen receptor directly) to be less effective among postmenopausal women with a high BMI [109]. The decreased efficacy is thought to be due to increased aromatization from adipose tissue of androstenedione to estrogens among overweight/obese women, resulting in insufficient suppression by standard treatment doses [33,109]. CLS-B detection and density might be a better marker of increased aromatization than BMI as a considerable proportion of ideal-weight women have CLS-B [71], and CLS-B are associated with increased aromatization in breast fat [80]. How CLS-B may influence the effectiveness of AIs has yet to be investigated.

The inflammatory potential of breast adipose tissue will be insufficiently quantified based on the presence or absence of CLS-B. Instead, focusing on CLS-B density may be one option for better determining the prognostic value of CLS-B. Combining indicators of inflammation such as evidence of local breast adipose tissue inflammation (as indicated by CLS-B and NF-κB binding activity in the breast tissue) and systemic inflammation (as indicated by elevated cytokines and chemokines in the blood) may be another way to identify women at particularly high risk of poor breast cancer outcomes. If so, these patients might benefit from lifestyle modification such as increased physical activity or treatment with pharmacologic agents such as non-steroidal anti-inflammatory drugs (NSAIDs), metformin, or statins that target obesity-related conditions and inflammation [110,111,112]. Findings from a recent preclinical rat model of ER-positive postmenopausal breast cancer showed that treatment with metformin decreased the size of existing mammary tumors and saw improvements in mammary tissue inflammation by decreases in CLS [113]. Additionally, these drugs target some of the same inflammatory pathways as CLS-B. For instance, certain statins are associated with deactivating NF-κB activity, which may sensitize tumor cells to chemotherapy and prevent chemoresistance [114,115]. There is currently no epidemiologic data that have examined the combined effects of local and systemic inflammation on breast cancer outcomes or whether pharmacologic agents influence the effects of CLS-B on breast cancer outcomes. However, randomized controlled trials assessing the effects of statins (e.g., NCT04601116), NSAIDs (e.g., NCT02927249), and metformin (e.g., NCT01101438), as well as lifestyle changes (e.g., NCT02786875) on breast cancer prognosis are already underway (ClinicalTrials.gov. Available online: www.clinicaltrials.gov) (accessed on 28 April 2021).

## 8. Conclusions

Obesity increases the incidence of postmenopausal HR+ tumors (and possibly premenopausal TNBC), and worsens prognosis, with limited evidence of differences by menopausal or HR status. The underlying mechanisms for these associations are not fully understood, but growing evidence points to the adipose tissue microenvironment playing an important role given the prevalence of adipose tissue in the breast and its metabolic and inflammatory activity. CLS-B are inflammatory lesions within the breast adipose tissue microenvironment associated with local NF-κB activation, increased aromatase activity, and elevation of pro-inflammatory mediators—all which could have numerous effects on breast cancer development and progression. While the evidence is continuing to accumulate, there is currently not enough epidemiologic data to answer key questions about the role of CLS-B in breast cancer etiology or prognosis overall or by molecular subtype. Interpretation of this evidence base is further complicated by small sample sizes, a lack of standardized methodology in CLS-B assessment, and other challenges such as tissue collection for assessment. Future epidemiologic studies should take these into consideration.

## Figures and Tables

**Table 1:** Overview of the epidemiologic studies examining CLS-B and female breast cancer.

First Author (Year)	Study Design	Institutions/ Affiliations	Country, Race/ Ethnicity Distribution	Study Population	Study Years	CLS-B Analyses Conducted
Breast cancer incidence studies (*n* = 2)						
Shaik (2020) [84]	Nested case–control + cross-sectional analysis	Detroit BBD cohort and KTB	USA 100% AA	*n* = 84 BBD cases *n* = 47 BBD controls *n* = 50 KTB volunteers without BBD or breast cancer	BBD diagnosis: 1997–2010 Follow up for breast cancer through 2016	Association between CLS-B and breast cancer among AA women with BBD CLS-B occurrence in normal breast tissue BMI and CLS-B associations Adipocyte diameter and CLS-B Associations between IL (another inflammatory marker) with breast cancer and BBD among AA women
Carter (2017) [85]	Nested case–control + cross-sectional analysis	Mayo BBD cohort and KTB	USA Unknown	*n* = 86 BBD cases *n* = 86 BBD controls *n* = 86 KTB volunteers without clinical breast abnormalities	BBD diagnosis: 1967–2001 Follow up for breast cancer: Unknown	Association between CLS-B and breast cancer among BBD patients CLS-B occurrence in normal breast tissue Participant and clinical characteristics associated with CLS-B Stromal CD68+ macrophage infiltration by BBD and breast cancer status
Breast cancer prognosis studies (*n* = 4)						
Maliniak (2020) [69]	Cohort + cross-sectional analysis	Emory University-affiliated tumor registries	USA 51% AA 49% White	*n* = 342 breast cancer patients Age ≥ 18 years old Stage I–III, invasive Underwent mastectomy No neoadjuvant treatment	Breast cancer diagnosis: 2007–2012 Follow up for breast cancer outcomes: 2018	Association between CLS-B and breast cancer prognosis Occurrence of CLS-B by race (AA vs. White) Participant and tumor characteristics associated with CLS-B Adipocyte number and CLS-B
Cha (2018) [86]	Cohort + cross-sectional analysis	Yonsei University	South Korea	Group 1: *n* = 56 non-breast cancer patients Tissue from reduction mammoplasty Group 2: *n* = 84 breast cancer patients Non-tumor breast tissue Group 3: *n* = 140 breast cancer patients Tumor-containing breast tissue	Unknown	Association between CLS-B and breast cancer prognosis (Group 3 only) Occurrence of CLS-B by breast cancer status and type of tissue among breast cancer patients (non-neoplastic vs. neoplastic) Participant and tumor characteristics associated with CLS-B (Group 3 only) Associations between number of infiltrating CD68+ and CD163+ in adipose tissue and tumor tissue with CLS-B detected by CD68 and CD163 (Group 3 only)
Koru-Sengul (2016) [87]	Cohort + cross-sectional analysis	University of Miami/Jackson Memorial Hospital tumor registry	USA 33% Black 33% non-Black Latina 33% Caucasian	*n* = 150 breast cancer patients Stage I–IV No previous exposure to chemotherapy, radiotherapy, or hormonal therapy	Cases obtained: 1978–1997 Followed for at least 5 years	Association between CLS-B and breast cancer prognosis Density of CLS-B across racial groups (Black, non-Black Latina, and Caucasian) Differences in densities of CLS-B macrophage phenotypes (M1, M2, pan) across racial groups Densities of TAMs by macrophage marker type and across racial groups Proliferative capacity of TAMs by proximity to tumor cells and across racial ethnic groups
Iyengar (2016) [82]	Cohort + cross-sectional analysis	MSKCC	USA 83% White 13% Black 3% Asian	Cohort 1: *n* = 100 patients (mostly breast cancer) Underwent mastectomy Cohort 2: *n* = 127 breast cancer patients All developed distant metastases but initially diagnosed with stage I–III breast cancer	Mastectomy: 2011–2013 (Cohort 1); 2001–2006 (Cohort 2) Cohort 2: Follow up for breast cancer outcomes: 2014	Cohort 1: Participant and tumor characteristics associated with CLS-B Circulating metabolic and inflammatory markers associated with CLS-B Cohort 2: Association between CLS-B and breast cancer prognosis Participant and tumor characteristics associated with CLS-B
Cross-sectional studies of CLS-B (*n* = 8)						
Greenlee (2018) [70]	Cross-sectional	Columbia University Medical Center	USA 100% Hispanic ^a^	*n* = 91 breast cancer patients Stage 0–III Underwent mastectomy 16% had neoadjuvant chemotherapy	Mastectomy: 2007–2012	Occurrence of CLS-B among Hispanic/Latina breast cancer patients Participant and tumor characteristics associated with CLS-B Adipocyte diameter and CLS-B Liver function biomarkers and CLS-B
Iyengar (2018) [81]	Cross-sectional	National Taiwan University Hospital and MSKCC	Taiwan USA 100% Caucasian	*n* = 72 Taiwanese breast cancer patients Non-metastatic Underwent mastectomy *n* = 267 US Caucasian patients Underwent mastectomy	Mastectomy: 2011–2016 (Taiwanese); 2011–2013 (US Caucasian)	Comparisons of CLS-B and breast adipocyte size in Taiwanese vs.US Caucasian women Participant and tumor characteristics associated with CLS-B (Taiwanese only) Body composition (body fat, VAT, SAT) factors associated with CLS-B (Taiwanese only) Circulating metabolic and inflammatory markers associated with CLS-B (Taiwanese only) Adipocyte diameter and CLS-B
Iyengar (2017) [71]	Cross-sectional	MSKCC	USA 76% Caucasian 9% Black, Asian, or Other 14% Unknown	*n* = 72 patients (mostly breast cancer) Normal weight (BMI < 25 kg/m^2^) Underwent mastectomy	Mastectomy: 2011–2013	Occurrence of CLS-B in normal weight women Participant and tumor characteristics associated with CLS-B Circulating metabolic and inflammatory markers associated with CLS-B Circulating leptin and CLS-B, aromatase expression, and adipocyte diameter associations Adipocyte diameter and CLS-B Aromatase activity and CLS-B, BMI, and adipocyte diameter associations
Mullooly (2017) [80]	Cross-sectional	PBCS	Poland	*n* = 83 breast cancer patients Invasive breast cancer Postmenopausal and not taking hormone replacement Underwent surgery No neoadjuvant treatment	Study recruitment: 2000–2003	Participant and tumor characteristics associated with CLS-B and number of CD68-positive cells CLS-B associations with concentrations and ratios of sex-steroid hormones in breast adipose tissue vs. systemic circulation
Vaysse (2017) [83]	Cross-sectional	Energy Balance and Breast Cancer Aspects-II	Norway	*n* = 107 breast cancer patients Aged 25–75 years Stage I–II, invasive 71% underwent breast conserving surgery	Unknown	BMI, WHR, % truncal fat and CLS-B associations overall and by menopausal status Circulating metabolic and inflammatory markers associated with CLS-B overall and by menopausal status Adipocyte diameter and CLS-B
Brown (2017) [72]	Cross-sectional	MSKCC	USA	*n* = 161 patients (mostly breast cancer) Aged 27–74 years Underwent mastectomy	Unknown	Menopause and CLS-B association The effect of menopause on CLS-B and aromatase expression associations
Iyengar (2015) [73]	Cross-sectional	MSKCC	USA 86% White 7% Black 6% Asian	*n* = 237 patients (mostly breast cancer) Aged 22–90 years Underwent mastectomy 14% received preoperative chemotherapy 39% of those tested had BRCA1/2 mutation	Mastectomy: 2011–2013	Participant and tumor characteristics associated with CLS-B Adipocyte diameter and CLS-B Comparison of CLS-B status between bilateral breasts Comparison of CLS-B status with abdominal CLS status
Morris (2011) [67]	Cross-sectional (pilot study)	MSKCC	USA	*n* = 30 patients (mostly breast cancer) Aged 26–70 years Underwent mastectomy	Enrolled: 2010	BMI and CLS-B association Adipocyte diameter and CLS-B Aromatase expression and activity and CLS-B NF-kB binding activity and CLS-B

Abbreviations: BBD = benign breast disease; BMI = body mass index; CLS-B = crown-like structures in breast adipose tissue; IL = infiltrating lymphocytes; KTB = Komen Normal Tissue Bank; MSKCC = Memorial Sloan Kettering Cancer Center; PBCS = Polish Breast Cancer Study; TAMs = tumor-associated macrophages; WHR = waist-to-hip ratio; USA = United States of America. ^a^ Greenlee et al. (2018) also included information on country of origin: 63% Dominican Republic, 16% Puerto Rican, 2% South American, 2% Mexican, and 16% Other Hispanic.

**Table 2:** Cross-sectional analyses examining the association between obesity and CLS-B.

First Author (Year)	Patient Study Population	% CLS-B+ by BMI (kg/m^2^) Group		Association between BMI and CLS-B aOR (95%CI) if Presented	Association with Other Adiposity Measures	Direction of Association: -/Null/+
Shaik (2020) [84]	BBD + Komen Normal Tissue Bank	NR		Not associated (*p* > 0.1)		Null
Carter (2017) [85]	BBD + Komen Normal Tissue Bank	BMI < 25: BMI 25–< 30: BMI ≥ 30:	7% 13% 29%			+
Maliniak (2020) [69]	Breast cancer	BMI < 25: BMI 25–< 30: BMI ≥ 30:	16% 29% 45%	Reference 2.34 (1.17 to 4.70) 4.73 (2.48 to 9.01)		+
Cha (2018) ^a^ [86]	Breast cancer	BMI < 25: BMI ≥ 25:	15% 27%			+
Greenlee (2018) [70]	Breast cancer	BMI 18.5–< 25: BMI 25–< 30: BMI 30–< 35: BMI ≥ 35:	24% 34% 57% 65%			+
Iyengar (2018) ^b^ [81]	Breast cancer	BMI < 23: BMI 23–< 27.5: BMI ≥ 27.5:	24% 48% 76%		Body fat, VAT, and SAT (all *p* < 0.01)	+
Iyengar (2017) [71]	Mostly breast cancer	All BMI < 25:	39%	CLS-B- vs. CLS-B + median BMI: 21.8 vs. 23.0, *p* = 0.04		+
Mullooly (2017) [80]	Breast cancer	BMI < 25: BMI 25–< 30: BMI ≥ 30:	17% 36% 54%	Reference 1.93 (0.50 to 7.40) 4.63 (1.08 to 19.83)		+
Vaysse (2017) [83]	Breast cancer	BMI < 25: BMI 25–< 30: BMI ≥30:	NR	Reference 3.2 (1.28 to 8.15) 6.9 (1.35 to 35.0)	WHR and % truncal fat (all *p* < 0.05)	+
Iyengar (2016) ^c^ [82]	Breast cancer	BMI < 25: BMI 25–< 30: BMI ≥ 30:	23% 33% 67%			+
Iyengar (2015) [73]	Mostly breast cancer	BMI < 25 BMI 25–< 30: BMI ≥ 30:	34% 53% 90%		CLS concordance between breast and abdominal SAT (*p* = 0.12)	+

Abbreviations: aOR = adjusted odds ratio; BBD = benign breast disease; BMI = body mass index; CLS-B = crown-like structures in the breast adipose tissue; NR = not reported; SAT = subcutaneous adipose tissue, VAT = visceral adipose tissue. ^a^ Cha et al. (2018) reported BMI associations for CLS-B detected using CD68 and CD163. The association for CLS detected by CD68 IHC is presented in the table for comparison with the other studies that all used CD68 for CLS-B detection. For CLS detected by CD163, % CLS-B was higher for women with BMI ≥ 25 (19%) compared BMI < 25 (11%). BMI associations were only reported for Group 3 (see Table 1). ^b^ Iyengar et al. (2018) [81] reports BMI associations for Taiwanese and US Caucasian patients. Associations for Taiwanese breast cancer patients are reported in the table above since presumably the US Caucasian subjects were included in the cohort from Iyengar et al. (2015) [73] which is already reported in the table. ^c^ Iyengar et al. (2016) [82] reports BMI associations for Cohorts 1 and 2. Associations for Cohort 2 are reported in the table above since the subjects in Cohort 1 are a sample of patients from Iyengar et al. (2015) [73] which is already reported in the table.

**Table 3:** Summary of the evidence between CLS-B and select patient and tumor characteristics.

First Author (Year)	N Studies	Summary of Evidence
Patient characteristics		
Obesity	11 studies [69,70,71,73,80,81,82,83,84,85,86]	Strong positive association in studies of breast cancer patients (see Table 2); inconclusive evidence for BBD patients and women without BBD or breast cancer
Age	8 studies [69,70,71,73,80,81,85,86]	Positive trend with age in studies of breast cancer patients although majority of associations were not statistically significant; no association observed between age and CLS-B among BBD patients [85]
Menopausal status	6 studies [69,70,71,72,73,81]	Positive trend with postmenopausal status among breast cancer patients although majority of associations were not statistically significant
Race/ethnicity	4 studies [69,70,81,87]	Evidence of greater CLS-B density among Black breast cancer patients in n = 2 studies [69,83] (no association when adjusting for BMI in the one study [69] with this information); No strong evidence of differences in CLS-B detection by country of origin among Hispanic/Latina patients [70] or when comparing Taiwanese to US Caucasian patients [80]
Smoking status	2 studies [69,70]	Positive trend with current smoking status in breast cancer patients but inconclusive (very few current smokers in both studies)
Age at menopause	2 studies [69,80]	Inconclusive evidence
Reproductive factors	2 studies [69,80]	Inconclusive evidence
Family history of breast cancer	2 studies [69,80]	Inconclusive evidence
Tumor characteristics		
Molecular subtype	6 studies [69,70,71,73,80,86]	No/little evidence for differences by ER status, PR status, or other tumor subtypes observed
Nodal status	4 studies [69,80,82,86]	Some evidence suggesting association with lymph node-negative disease but all together inconclusive
Grade	4 studies [69,80,82,86]	Inconclusive evidence
Stage	3 studies [69,70,86]	Inconclusive evidence

Abbreviations: BBD = benign breast disease; CLS-B = crown-like structures in the breast adipose tissue; ER = estrogen receptor; PR = progesterone receptor.

**Table 4:** Epidemiologic evidence examining CLS-B as a potential driver of female breast cancer incidence and prognosis.

First Author (Year)	N Total	Study Design	Antibody: % CLS-B+	Outcome	N Outcomes	Adjusted Estimate (95%CI) if Reported	Summary of Results Caveats
Breast cancer incidence studies (*n* = 2)							
Shaik (2020) [84]	55 cases/ 47 controls	Nested case–control	CD68: Cases: 67% Controls: 40%	Invasive breast cancer	-	Any CLS-B vs. none: 3.98 (1.40 to 11.3) ≥5 CLS-B/sample vs. none: 4.99 (1.32 to 18.9)	Positive association between CLS-B and breast cancer among BBD patients Wide CI
Carter (2017) [85]	86 cases / 86 controls	Nested case–control	CD68: Cases: 24% Controls: 19%	Invasive or in situ breast cancer	-	Any CLS-B vs. none: NR >5 CLS-B/sample vs. none: 6.8 (1.4 to 32.4)	Positive association between CLS-B and breast cancer among BBD patients Wide CI
Breast cancer prognosis studies (*n* = 4)							
Maliniak (2020) [69]	319	Cohort	CD68: 30%	OS PFS	46 recurrences 52 deaths	OS (Any CLS-B vs. none): 1.02 (0.55 to 1.87) PFS (Any CLS-B vs. none): 0.99 (0.59 to 1.67)	Null association between CLS-B and breast cancer prognosis in a diverse population of breast cancer patients Wide CI Possible misclassification of CLS-B
Cha (2018) [86]	140 ^a^	Cohort	CD68: 18% CD163: 13%	OS DFS	18 recurrences 11 deaths	OS (CLS-B present vs. absent): CD68: univariate *p* = 0.390 CD163 univariate *p* = 0.492 DFS (CLS-B present vs. absent): CD68: univariate *p* = 0.899 CD163: univariate *p* = 0.883	Not enough breast cancer outcomes to draw conclusions
Koru-Sengul (2016) [87]	150	Cohort	CD163: NR CD40: NR CD206: NR	OS PFS	83 recurrences 88 deaths	OS (density of CLS): CD163: 2.14 (0.46 to 9.96) ^b^ CD40: 9.14 (1.00 to 83.60) ^b^ CD206: 0.65 (0.03 to 12.58) ^b^ PFS (density of CLS): CD163: 2.30 (0.66 to 8.03) ^b^ CD40: 4.12 (0.49 to 34.92) ^b^ CD206: 1.16 (0.09 to 14.28) ^b^	Positive association between CLS-B and breast cancer prognosis Wide CI Varied by antibody used for detecting CLS-B Assessed CLS-B in tumor tissue
Iyengar (2016) [82]	127	Case-only analysis	CD68: 41%	Average time to distant recurrence	127 recurrences 99 deaths	Any CLS-B vs. none: 1.83 (1.07 to 3.13)	Positive association between CLS-B and breast cancer prognosis Select population of patients that all developed metastatic disease

NOTE: factors adjusted for varied in different analyses. Abbreviations: CI = confidence interval; CLS-B = crown-like structures in the breast adipose tissue; DFS = disease-free survival; NR = not reported; OS = overall survival; PFS = progression-free survival. ^a^ Cha et al. (2018) [86] only reported OS and DFS associations for Group 3 (see Table 1). ^b^ 90% CIs reported.

**Table 5:** Summary of CLS-B assessment methods by epidemiologic study.

First Author (Year)	Tissue Specimen	Tissue Specimens per Subject	Antibody	% CLS-B+
Breast cancer incidence studies (*n* = 2)				
Shaik (2020) [84]	BBD: FFPE BBD biopsy tissue KTB donors: FFPE percutaneous needle biopsy tissue	1	CD68	BBD Cases: 67% BBD Controls: 40% KTB donors: 18%
Carter (2017) [85]	BBD: FFPE BBD biopsy tissue KTB donors: FFPE normal breast tissue	1	CD68	BBD Cases: 24% BBD Controls: 19% KTB donors: 3%
Breast cancer prognosis studies (*n* = 4)				
Maliniak (2020) [69]	FFPE non-tumor tissue	1	CD68	Overall: 30% AA: 32% White: 29%
Cha (2018) [86]	Group 1: FFPE reduction mammoplasty Group 2: FFPE non-tumor tissue Group 3: FFPE tumor tissue	Unknown	CD68 CD163	CD68, CD163 Group 1: 2%, 2% Group 2: 0%, 0% Group 3: 18%, 13%
Koru-Sengul (2016) [87]	FFPE tumor tissue	1	CD163 CD206 CD40	Density of CLS: CD163, CD206, CD40 Mean (SD) All: 0.06 (0.14); 0.03 (0.07); 0.01 (0.07) Black: 0.11 (0.22); 0.04 (0.09); 0.02 (0.11) NBLA: 0.05 (0.08); 0.03 (0.05); 0 (0) CA: 0.03 (0.07); 0.02 (0.06); 0 (0.02)
Iyengar (2016) [82]	FFPE non-tumor tissue	5	CD68	41%
Cross-sectional studies of CLS-B (*n* = 8)				
Greenlee (2018) [70]	FFPE non-tumor tissue	5	CD68	45%
Iyengar (2018) [81]	FFPE non-tumor tissue	5	CD68	Taiwanese: 43% US Caucasian: 55%
Iyengar (2017) [71]	FFPE non-tumor tissue	5	CD68	39%
Mullooly (2017) [80]	FFPE non-tumor tissue	1	CD68	36%
Vaysse (2017) [83]	FFPE tumor tissue	Unknown	CD68	54%
Brown (2017) [72]	FFPE non-tumor tissue	5	CD68	57%
Iyengar (2015) [73]	FFPE non-tumor tissue	5	CD68	51%
Morris (2011) [67]	FFPE non-tumor tissue	4–5	CD68	47%

Abbreviations: AA = African American; BBD = benign breast disease; CA = Caucasian; CLS-B = crown-like structures in breast adipose tissue; FFPE = formalin-fixed paraffin-embedded; KTB = Komen Normal Tissue Bank; NBLA = non-Black Latina; US = United States.

## Data Availability

No new data were generated or analyzed in support of this research.
